# Infant gross motor development and childhood physical activity: Role of adiposity

**DOI:** 10.1016/j.jsampl.2023.100021

**Published:** 2023-01-23

**Authors:** Tomoko Aoyama, Yuki Hikihara, Masashi Watanabe, Hitoshi Wakabayashi, Satoshi Hanawa, Naomi Omi, Hidemi Takimoto, Shigeho Tanaka

**Affiliations:** a Department of Nutritional Epidemiology and Shokuiku, National Institutes of Biomedical Innovation, Health and Nutrition, 1-23-1 Toyama, Shinjuku-ku, Tokyo 162-8636 Japan; b Advanced Research Center for Human Sciences, Waseda University, 2-579-15 Mikajima, Tokorozawa-shi, Saitama 359-1192, Japan; c Faculty of Creative Engineering, Center for Liberal Arts, Chiba Institute of Technology, 2-1-1 Shibazono, Narashino-shi, Chiba 275-0023 Japan; d College of Education, Ibaraki University, 2-1-1 Bunkyo, Mito-shi, Ibaraki 310-8512 Japan; e Faculty of Engineering, Hokkaido University, Kita 13, Nishi 8, Kita-ku, Sapporo-shi, Hokkaido 060-8628 Japan; f Meiji Yasuda Life Foundation of Health and Welfare, 8-3, Nishisinjuku, Shinjuku-ku, Tokyo 160-0023 Japan; g Faculty of Health and Sport Sciences, University of Tsukuba, 1-1-1 Tennodai, Tukuba-shi, Ibaraki 305-8577 Japan; h Department of Nutritional Science, National Institutes of Biomedical Innovation, Health and Nutrition, 1-23-1 Toyama, Shinjuku-ku, Tokyo 162-8636 Japan; i Faculty of Nutrition, Kagawa Nutrition University, 3-9-21 Chiyoda, Sakado-shi, Saitama 350-0288 Japan

**Keywords:** Growth and development, Motor skills, Infant, Child, Physical activity, Accelerometer

## Abstract

**Objectives:**

Later achievement of standing and walking in infancy predicts childhood physical inactivity. This study aimed to examine associations between ages of achieving six gross motor milestones and childhood physical activity, and whether these associations were mediated by adiposity.

**Design:**

A retrospective analysis of a subset from a cohort study.

**Methods:**

Data were available for 211 first-grade primary school children (aged 6–7 years) in the Kanto region, Japan. Information on ages of achieving holding head up, sitting, crawling, standing supported, walking supported, and independent walking were obtained from parental records in the Maternal and Child Health Handbooks. Adiposity was assessed using dual-energy X-ray absorptiometry, and expressed as body fat percentage. Current gross motor skills were assessed by the Test of Gross Motor Development 2nd edition. Physical activity was measured using a triaxial accelerometer and defined as time involved in moderate-to-vigorous-intensity physical activity (MVPA).

**Results:**

Multiple regression analyses revealed that the age of achieving standing supported was negatively associated with MVPA (p ​= ​.021), while ages of achieving crawling (p ​= ​.010), standing supported (p ​= ​.002), and walking supported (p ​= ​.033) were positively associated with adiposity, after adjusting for potential confounders including current gross motor skills. When adiposity was introduced as a covariate, the age of achieving standing supported was still associated with MVPA (p ​= ​.048), and the indirect effect of achievement of standing supported on MVPA was not significant.

**Conclusions:**

Infants who achieve standing supported at a later age are less likely to be active during early school age, and adiposity may not mediate this association.

## Abbreviations

BMI: body mass index
DXA: dual-energy X-ray absorptiometry
%Fat: percentage of body fat
MCHH: Maternal and Child Health Handbook
MVPA: moderate-to-vigorous-intensity physical activity
TGMD2: Test of Gross Motor Development 2nd edition

## 1 Introduction

Childhood physical activity is associated with a wide range of health benefits, including improved physical fitness, cardiovascular metabolic health, bone health, cognitive outcomes, and mental health [1]. Nevertheless, many children and adolescents do not meet the current guideline-based recommendations [2,3] that suggest they should engage in moderate-to-vigorous-intensity physical activity (MVPA) for ≥60 ​min/day [1]. Since physical inactivity begins to take hold early in life [4], childhood physical activity potentially has a lifelong impact, which in turn can affect the risk of death and non-communicable diseases. Hence, increasing current physical activity among children is an urgent public health issue, and is also important for reducing future public health burdens. Gaining a better understanding of the determinants of physical activity during childhood is important to develop effective strategies to promote physical activity across the lifespan.

Recent evidence suggests potential contributions of early-life factors to young people's physical activity [5,6]. Poor motor coordination at 6 months of age [7] and later ages of achieving standing and walking [8] were associated with lower levels of physical activity in young people in their early teenage years. Sanchez et al. found that gross motor coordination, but not fine motor coordination, at 9 months of age was associated with time involved in MVPA (MVPA time) at 7 years of age [9]. More recently, other investigators have demonstrated associations between later achievement of walking and less MVPA time in preschool [10] and primary school [11] children. Hence, the age at which gross motor milestones are achieved in infancy can be an early-life predictor of physical activity status during childhood. However, the association of gross motor milestones other than standing and walking with childhood physical activity remains unclear.

Gross motor development also predicts childhood adiposity. Later achievement of rolling over, sitting, and walking was associated with increased adiposity levels in children aged 3 years [12]. We previously reported that later achievement of crawling, standing supported, and walking supported can predict adiposity measured by dual-energy X-ray absorptiometry (DXA) in children aged 6–7 years [13]. Adiposity often occurs in conjunction with physical inactivity [14]. However, results from a Mendelian randomised analysis of 11-year-old children [15] and a longitudinal observational study of children aged 7–10 years [16] indicated that while adiposity causes physical inactivity, physical inactivity does not cause increased adiposity during childhood. Thus, adiposity might mediate associations between infant gross motor development and childhood physical activity. Understanding the role of adiposity in the association between infant gross motor development and childhood physical activity can lead to appropriate interventions to prevent physical inactivity early in life.

Therefore, this study aimed to examine associations between ages of achieving different gross motor developmental milestones in infancy and physical activity during childhood, and to investigate whether these associations were mediated by adiposity during childhood.

## 2 Methods

This study was a secondary analysis using a subset of data from our previous study [13], which investigated gross motor milestone achievement and childhood adiposity. In summary, 248 first-grade children, comprising boys (n ​= ​153) and girls (n ​= ​95) aged 6–7 years, had participated in the original study [13]. The participants were recruited through five primary schools across three prefectures (Tokyo, Chiba, and Ibaraki) in the Kanto region, Japan. The children and their parents visited either the National Institute of Health and Nutrition or the University of Tsukuba for body composition and motor competence [17] measurements between July and September in 2012 or 2013, followed by daily physical activity assessments using accelerometers on 14 consecutive days between the months of October and November during the school term. Of the 248 participants, Maternal and Child Health Handbooks (MCHH) data were collected from 240 participants at the time of visit. The MCHH is a home-based health record issued by local governments when a woman registers her pregnancy in Japan. This handbook contains records of pregnancy checkups, delivery, and health checkups and immunisations for children from birth to school ages, provided by health professionals [18]. It also contains sections for pregnant women to report pre-pregnancy conditions and for parents to record their child's development. The previous study was based on 225 participants, after excluding those with very low birth weights (<1.5 ​kg) (n ​= ​1) and missing all data on gross motor development (n ​= ​14). From the data of 225 participants in the previous study, we excluded 14 participants whose accelerometer data did not conform to the inclusion criteria of this study (as described later). The final dataset included 211 children (126 boys and 85 girls).

At the visit, body height and weight were measured to the nearest 0.1 ​cm and 0.1 ​kg, respectively, with the children wearing light underwear and no shoes. Body mass index (BMI) was calculated in kg/m^2^ from height and weight. Percentage of body fat (%Fat) was estimated using DXA (Hologic QDR-4500; Hologic, Inc., Waltham, MA, USA). Current gross motor skills were also assessed by the Test of Gross Motor Development 2nd edition (TGMD2) [17]. This test is designed for children aged 3–10 years based on observations of movements across 12 tasks including locomotor (run, gallop, hop, leap, horizontal jump, and slide) and object control (striking a stationary ball, stationary dribble, catch, kick, overhand throw, and underhand roll) skills [19]. In the analyses, the sum of the scores for the 12 tasks was used as an indicator of gross motor skills and expressed as the TGMD2 score.

Physical activity was measured for 14 consecutive days using triaxial accelerometers (Active style Pro HJA-350IT; Omron Healthcare, Kyoto, Japan) [17]. Details of the device were previously described [20,21]. The children were asked to wear the accelerometers on their waists constantly, except during sleeping, bathing, and swimming. Ten-second epochs were used, and the data were converted for primary school children using the following equations [20]:

Locomotive activities: 0.6237 ​× ​metabolic equivalents value ​+ ​0.2411.

Non-locomotive activities: 0.6145 ​× ​metabolic equivalents value ​+ ​0.5573.

Locomotive activities (walking and running) and non-locomotive activities (playing games, cleaning, playing with blocks or a ball, and aerobic dance, etc.) were distinguished based on the ratios of unfiltered synthetic acceleration to filtered synthetic acceleration [20]. We analysed data collected between 7:00 AM and 9:00 PM, which excluded any data gathered while the children slept [11]. Periods of consecutive zero counts that indicate no signal for <20 ​min were incorporated into ‘accelerometer wear time’, and children with accelerometer wear times of >10 ​h on ≥5 weekdays and ≥2 days on the weekends [22] were included in the analysis to ensure the minimum criterion of ≥4–5 days for school-aged children [23]. The mean MVPA time in min/day (≥3.0 metabolic equivalents value, predicted based on measured resting metabolic rate [20]) was calculated by weighting the data for 5 weekdays and 2 ​days ​at the weekends as follows: weighted data = ([mean for weekdays ​× ​5] ​+ ​[mean for weekend days ​× ​2])／7.

Data from MCHHs were transcribed to a questionnaire by parents [13]. Information on child development such as date on or age at which the children achieved six gross motor milestones was obtained from parental records in the MCHHs. The six gross motor milestones included holding head up, sitting without support (sitting), hands-and-knees crawling (crawling), standing supported, walking supported, and independent walking. Information on potential confounders such as maternal height and pre-pregnancy weight [24] (self-reported), maternal age and gestational age at delivery, and birth weight [[5], [6]] were also collected from the MCHHs. We then calculated maternal pre-pregnancy BMI (kg/m^2^) and the ages (months) at which the children achieved the six gross motor milestones. Some children had missing information on maternal BMI (n = 2) and ages at which gross motor milestones were achieved (the available number for each milestone is shown in the tables), as these were recorded at the discretion of the mother or parents. Further, the child's birth order was reported by the parents using the questionnaire.

The measured and calculated values were presented as means and standard deviations (SDs). Partial correlation analyses were used to evaluate relationships between early predictors, %Fat, TGMD2 score, and MVPA time, controlled for sex as a covariate. Multiple regression analyses were performed to assess associations of ages of achieving six gross motor milestones with %Fat and MVPA time, after adjusting for sex, maternal age and gestational age at delivery, birth order, school location (prefecture), TGMD2 score, and accelerometer wear time (in the model predicting MVPA). Subsequently, to determine whether adiposity mediates the association between gross motor development and MVPA time, we included %Fat as a covariate in the model predicting MVPA time, using the methods described by Baron and Kenny [25]. We also considered the influence of other potential confounders (i.e., maternal pre-pregnancy BMI and birth weight) on the model, however, there was no strong evidence of associations between these factors and MVPA time, and thus, they were not included in the final model. Statistical analyses were performed using IBM®SPSS® software, version 28.0 for Windows (IBM Ltd., Armonk, NY, USA). Statistical significance was set at p ​< ​.05. Mediation analysis was performed by the PROCESS macro for SPSS version 4.2, with bootstrapping methods [26].

## 3 Results

Table 1 shows the participants’ characteristics. The mean number of days on which the accelerometers were worn was 12.4 (SD 1.7) days. The mean time involved in MVPA was 81.1 (SD 22.5) min/day, and 18% (n ​= ​38) of the children did not engage in MVPA for ≥60 ​min/day. Non-locomotive activities accounted for nearly half (44%) of the total MVPA. Compared with the girls, the boys spent more time performing MVPA (95% confidence interval [CI]: 13.9, 25.2), including both locomotive (95% CI: 11.3, 19.3) and non-locomotive (95% CI: 1.7, 6.8) MVPA.

**Table 1:** Characteristics of the study participants.

Variables	Mean (SD) or number (%)	Range
Age (months)	82.9 (3.5)	76–89
Height (cm)	118.8 (4.8)	107.1–130.5
Weight (kg)	21.5 (2.9)	16.1–33.4
BMI (kg/m^2^)	15.2 (1.4)	12.8–22.3
%Fat (%)	21.8 (4.6)	13.1–37.2
TGMD2 score	73.3 (10.6)	42–93
MVPA (min/day)	81.1 (22.5)	33.5–168.8
Locomotive MVPA (min/day)	45.8 (17.2)	16.9–122.1
Non-locomotive MVPA (min/day)	35.3 (9.5)	15.1–60.8
Accelerometer wear time (min/day)	772 (27)	648–835
**Perinatal**		
Maternal pre-pregnancy BMI (kg/m^2^)	20.4 (2.8)	16.1–35.4
Maternal age at delivery (years)	31.9 (4.6)	21–43
Gestational age at delivery (weeks)	38.9 (1.5)	32–41
Birth weight (g)	3000 (404)	1614–4064
Birth order		
1	142 (67)	
2	54 (26)	
>2	15 (7)	
**Age of achieving (months)**		
Holding head up (n ​= ​127)	3.3 (0.6)	1.4–5.4
Sitting (n ​= ​99)	6.6 (0.8)	4.8–9.0
Crawling (n ​= ​150)	8.0 (1.5)	3.8–12.6
Standing supported (n ​= ​147)	8.3 (1.1)	6.2–12.1
Walking supported (n ​= ​118)	9.4 (1.5)	6.5–13.9
Independent walking (n ​= ​179)	12.8 (2.0)	9.0–19.0

BMI, body mass index; Crawling, hands-and-knees crawling; MVPA, moderate-to-vigorous-intensity physical activity; SD, standard deviations; Sitting, sitting without support; TGMD2, Test of Gross Motor Development 2nd edition; %Fat, percentage of body fat.

Partial correlations analyses controlled for sex (Supplementary Table A.1) showed that %Fat, TGMD2 score, and MVPA time were correlated with each other (p ​< ​.001). The ages of achieving four milestones were negatively correlated with MVPA time (p ​< ​.05), and three milestones were positively correlated with %Fat (p ​< ​.05). However, none of the six milestones were correlated with TGMD2 score. Multiple regression analyses (Table 2) revealed that the age of achieving standing supported was negatively associated with MVPA time (p ​= ​.021), even after adjustment for confounders including TGMD2 score. The ages of achieving crawling (p ​= ​.010), standing supported (p ​= ​.002), and walking supported (p ​= ​.033) were positively associated with %Fat, as previously reported [13].

**Table 2:** Multiple regression analyses for the associations of gross motor development with childhood physical activity and adiposity.

Age of achieving (months)	n	MVPA (min/day)				%Fat (%)			
		B	β	(95% CI)	p	B	β	(95% CI)	p
Holding head up	127	0.46	.01	(−5.2, 6.2)	.874	0.96	.13	(−0.2, 2.1)	.097
Sitting	99	2.58	.09	(−2.4, 7.6)	.308	0.30	.06	(−0.6, 1.2)	.516
Crawling	150	−1.94	−.12	(−4.2, 0.3)	.090	**0.59**	**.18**	**(0.1, 1.0)**	**.010**
Standing supported	147	**−3.60**	**−.17**	**(−6.6, −0.6)**	**.021**	**0.90**	**.22**	**(0.3, 1.5)**	**.002**
Walking supported	118	−1.82	−.12	(−4.5, 0.9)	.188	**0.56**	**.18**	**(0.05, 1.1)**	**.033**
Independent walking	179	−0.82	−.07	(−2.3, 0.7)	.290	0.01	.002	(−0.3, 0.3)	.972

Adjusted for sex, maternal age and gestational age at delivery, birth order, school location, TGMD2 score, and accelerometer wear time (in the model predicting MVPA).

B, unstandardised regression coefficient; β, standardised regression coefficient; CI, confidence interval.

Crawling, hands-and-knees crawling; MVPA, moderate-to-vigorous-intensity physical activity; Sitting, sitting without support; TGMD2, Test of Gross Motor Development 2nd edition; %Fat, percentage of body fat.

**Fig. 1:**
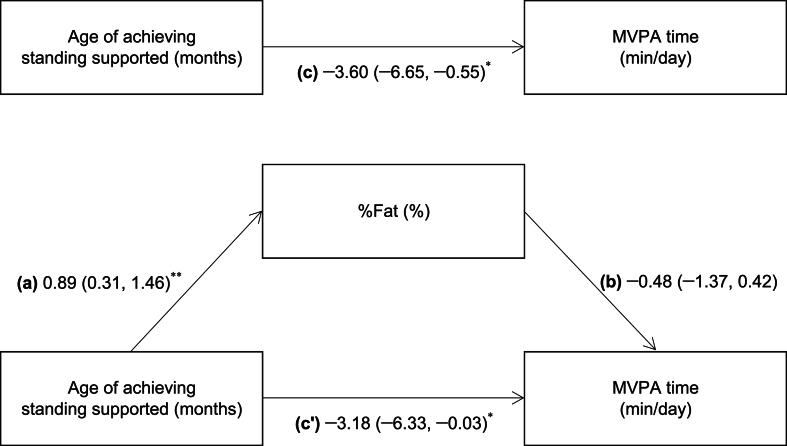
Effect of the age of achieving standing supported on MVPA time, without considering mediation (upper) and including mediation (lower) by adiposity Data are unstandardised regression coefficients (95% confidence intervals). ∗p < .05, ∗∗p < .01. All models were adjusted for sex, maternal age and gestational age at delivery, birth order, school location, TGMD2 score, and accelerometer wear time. The paths represent the difference in %Fat (%) per 1-month increase in the age of achieving standing supported (a), difference in MVPA time (min/day) per 1% increase in %Fat (b), and differences in MVPA time (min/day) per 1-month increase in the age of achieving standing supported, with (c’) and without (c) adjustment for %Fat. Total effect (c) = c’ + ab; Indirect effect (ab) = c – c’; Direct effect (c’) = c – ab. MVPA, moderate-to-vigorous-intensity physical activity; TGMD2, Test of Gross Motor Development 2nd edition; %Fat, percentage of body fat.

Finally, when %Fat was introduced in the model predicting MVPA (Fig. 1), the association between the age of achieving standing supported and MVPA time remained significant (p ​= ​.048), while %Fat was not significantly associated with MVPA time (p ​= ​.295). The indirect effect of the age of achieving standing supported on MVPA time was not significant (a ​× ​b path; B ​= ​−0.42, 95% CI: −1.43, 0.35).

## 4 Discussion

This study aimed to investigate associations between the ages of achieving six gross motor milestones and physical activity in children, and to determine whether these associations were mediated by adiposity. In the partial correlation analysis, consistent negative correlations were observed between ages of achieving gross motor milestones (with the exception of sitting) and MVPA time, though not all of these correlations were statistically significant and the degree of correlation was weak. In principal, later gross motor development appears to set children in the direction of being inactive. However, in multiple regression analysis with adjustment for potential confounders including current gross motor skills determined by TGMD2, a significant association was only detected between the age of achieving standing supported and MVPA time. Previous studies had only examined the association of one or two milestones, such as standing unaided [8] and walking supported [8] or independent walking [[10], [11]] with childhood physical activity, whereas this study examined six different milestones. Our finding on standing supported was generally consistent with the findings of Ridgway et al. [8], in which a questionnaire was used to assess physical activity in terms of the frequencies of sports participation. The present study is the first to show the link between ages of achieving gross motor milestones other than walking [[10], [11]] and ‘objectively’ measured MVPA. Achieving standing supported (approximately 8 months of age), which is usually observed earlier than achieving independent walking (approximately 12 months of age) [27], can predict MVPA during early school age.

Crawling develops at approximately the same time (8 months of age) as standing supported. Nevertheless, only the age of achieving standing supported was associated with MVPA time. This might be related to the nature of the two activities; While standing supported is a ‘posture’ that can be easily recognised after it has been achieved, crawling is a ‘locomotion’ that can be perceived differently by each parent. There may be variations in the definition of how far an infant has to travel on hands and knees to be considered to have achieved crawling. Further, while crawling is not observed in 4% of infants (i.e., non-crawler), all infants achieve standing supported before the onset of walking [27]. Therefore, among the six milestones, standing supported seems to be the most practical milestone to observe for predicting child health including adiposity and physical inactivity.

In this study sample, each 1-month increase in a child's age of achieving standing supported resulted in a 3.6 ​min/day decrease in MVPA (Table 2). Given that the age at which infants first show standing supported are generally distributed over 6.6 months [27], individual differences in such gross motor development may explain up to approximately 24 ​min/day difference in MVPA during early school age, which corresponds almost 1 SD of MVPA time in this study sample. While infant gross motor development is multifactorial [28], randomised control trials have demonstrated that this process is modifiable [29,30]. Therefore, there is scope for interventions to promote gross motor development. Although this study could not demonstrate a causal relationship, promoting early gross motor development could have a positive long-term impact on lifelong physical activity if it could prevent childhood physical inactivity.

Unlike findings from previous studies [8,10,11], the associations of the ages of achieving walking supported and independent walking with MVPA time were not significant. This discrepancy might be related, in part, to our small sample size as compared with those of previous studies. Interestingly, when MVPA was divided into locomotive and non-locomotive activities, the ages of achieving walking supported and independent walking showed clear associations with non-locomotive MVPA (Supplementary Table A.2). As children's habitual physical activity behaviours are more complex than those of adults, distinguishing between locomotive and non-locomotive activities when measuring physical activity contributes to accurate estimates of MVPA in children [[20], [21]]. Our supplementary analysis was performed using this discrimination. Although the cause of the association between walking development and ‘non-locomotive’ MVPA remains unclear, the associations between motor development and childhood MVPA (and its components) seem to differ by the type of gross motor milestone, with walking development having a unique relationship with non-locomotive activity. Further investigation is necessary to clarify this point.

We did not find any associations between the ages of achieving holding head up and sitting with MVPA time. Although moderate correlations existed among the ages of achieving the six milestones [13], holding head up and sitting have relatively short timeframes in which these milestones should be accomplished. This might explain the absence of their associations with MVPA time. In this study sample, holding head up and sitting were accomplished in approximately 4 months, while other gross milestones were achieved in 6–12 months (Table 1).

In the mediation analysis, %Fat did not mediate the relationship between the age of achieving standing supported and MVPA time. This observation also means that the age of achieving standing supported may predict childhood physical activity, independent of adiposity. At the same time, childhood adiposity may not explain the association between the achievement of standing supported and physical activity, suggesting that unmeasured factors may mediate this association. Enjoyment may be one of the possible factors, through which gross motor development impacts long-term physical activity [31]. Further studies are needed to identify factors that can mediate the associations between gross motor development during infancy and childhood physical activity.

This study has several limitations. First, we used MCHH records to obtain data describing the dates on or ages at which the six gross motor milestones were achieved. While parental assessments of their children's gross motor milestone achievements are fairly reliable [32], the dates and ages recorded in the MCHH were at the discretion of the parents, and thus, some data were missing. Therefore, an unknown bias derived from the missing data may have affected the results. Second, as a considerable amount of data were missing, we could not evaluate the sequences or specific patterns in which the milestones were achieved. Further studies are needed to investigate these issues. Third, since this was an exploratory and retrospective study with a small subset sample, the findings are preliminary. Larger follow-up studies from birth are needed to establish the associations between gross motor development, adiposity, and physical activity. While these limitations prompt caution when interpreting the findings, this study has some strengths including precise measurements of physical activity and adiposity using accelerometers and DXA, respectively, with consideration of current gross motor skills. These measurements will help us to understand the mechanisms linking gross motor development during infancy to childhood physical activity.

## 5 Conclusions

This study showed that children who achieved standing supported at a later age in infancy spent less time in MVPA during early school age. Furthermore, adiposity did not mediate this association. This study suggests the need for further research, such as follow-up studies in birth cohorts, to clarify how physical inactivity is determined in relation to gross motor development and adiposity early in life, and to develop effective measures to promote lifelong physical activity.

## 6 Practical implications

● Infants who were older when they first showed standing supported are less likely to be active during early school age.
● Observation of gross motor milestone achievement, such as standing supported, might help identify infants with possible need of early interventions to prevent childhood physical inactivity.
● There was no strong evidence indicating that optimising body composition is an effective means of such early intervention.

## Funding information

This work was supported by 10.13039/501100001691JSPS KAKENHI (grant numbers JP13J07359, JP20K13947, JP24700759, and JP25750373) and the Yamaha Motor Foundation for Sports. The funders had no role in study design, data collection and analysis, decision to publish, or preparation of the manuscript.

## Confirmation of ethical compliance

The study was approved by the ethics committee of the National Institute of Health and Nutrition (20121012–01), and conformed with the Declaration of Helsinki. The study procedures were explained in writing to all the children and their parents, and written informed consent was obtained from the parents.

## Declaration of competing interest

The authors declare that they have no known competing financial interests or personal relationships that could have appeared to influence the work reported in this paper.
