# Supplementary material for: Infant gross motor development and childhood physical activity: Role of adiposity
Source: JSAMS Plus. 2023 Jan 23;2:100021. doi: 10.1016/j.jsampl.2023.100021 (PMC13008445; doi:10.1016/j.jsampl.2023.100021)
Supplement: Multimedia component 1 [file mmc1.docx]

**Supplementary Table A.1**

Partial correlations between early-life factors and childhood adiposity, gross motor skills, and physical activity (controlled for sex).

| **Variables** | n | **TGMD2 score** | **%Fat (%)** | **MVPA (min/day)** | **Locomotive MVPA (min/day)** | **Non-locomotive MVPA (min/day)** |
| --- | --- | --- | --- | --- | --- | --- |
| Maternal pre-pregnancy BMI (kg/m^2^) | 209 | −.08 | **.23***** | .02 | −.02 | .07 |
| Maternal age at delivery (years) | 211 | −.11 | .01 | **−.15*** | −.07 | **−.20**** |
| Gestational age at delivery (weeks) | 211 | .01 | −.05 | .06 | .04 | .06 |
| Birth weight (g) | 211 | .06 | −.005 | −.02 | −.06 | .05 |
| Birth order | 211 | .11 | −.09 | .07 | .01 | **.15*** |
| **Age of achieving (months)** |  |  |  |  |  |  |
| Holding head up | 127 | −.04 | .15 | −.02 | −.06 | .06 |
| Sitting | 99 | −.12 | .08 | .02 | .06 | −.06 |
| Crawling | 150 | −.13 | **.26**** | **−.19*** | −.15 | −.15 |
| Standing supported | 147 | −.12 | **.29***** | **−.21**** | **−.17*** | **−.19*** |
| Walking supported | 118 | −.09 | **.21*** | **−.19*** | −.09 | **−.26**** |
| Independent walking | 179 | −.10 | **.**06 | **−.15*** | −.06 | **−.22**** |
| TGMD2 score | 211 |  | **−.32***** | **.35***** | **.33***** | **.21**** |
| %Fat (%) | 211 |  |  | **−.23***** | **−.19**** | **−.18**** |
| MVPA (min/day) | 211 |  |  |  | **.90***** | **.69***** |
| Locomotive MVPA (min/day) | 211 |  |  |  |  | **.31***** |

*p <.05; **p <.01; ***p <.001.

BMI, body mass index; Crawling, hands-and-knees crawling; MVPA, moderate-to-vigorous-intensity physical activity; Sitting, sitting without support; TGMD 2, Test of Gross Motor Development 2nd edition; %Fat, percentage of body fat.

**Supplementary Table A.2**

Multiple regression analyses for the associations between gross motor development and locomotive and non-locomotive physical activity.

| **Age of achieving  (months)** | n | **Locomotive MVPA (min/day)** | | | |  | **Non-locomotive MVPA (min/day)** | | | |
| --- | --- | --- | --- | --- | --- | --- | --- | --- | --- | --- |
|  |  | B | β | (95% CI) | p |  | B | β | (95% CI) | p |
| Holding head up | 127 | −0.30 | −.01 | (−4.8, 4.2) | .894 |  | 0.76 | .05 | (−1.8, 3.3) | .556 |
| Sitting | 99 | 2.45 | .11 | (−1.6, 6.5) | .227 |  | 0.13 | .01 | (−1.9, 2.2) | .899 |
| Crawling | 150 | −1.19 | −.10 | (−3.0, 0.6) | .186 |  | −0.75 | −.12 | (−1.7, 0.2) | .131 |
| Standing supported | 147 | −2.21 | −.14 | (−4.6, 0.2) | .067 |  | **−1.39** | **−.17** | **(−2.7, −0.1)** | **.040** |
| Walking supported | 118 | −0.49 | −.04 | (−2.6, 1.6) | .647 |  | **−1.32** | **−.20** | **(−2.5, −0.1)** | **.029** |
| Independent walking | 179 | −0.02 | −.002 | (−1.2, 1.1) | .977 |  | **−0.80** | **−.17** | **(−1.5, −0.1)** | **.019** |

Adjusted for sex, maternal age and gestational age at delivery, birth order, school location, TGMD2 score, and accelerometer wear time.

B, non-standardised regression coefficient; β, standardised regression coefficient; CI, confidence interval; Crawling, hands-and-knees crawling; MVPA, moderate-to-vigorous-intensity physical activity; Sitting, sitting without support; TGMD2, Test of Gross Motor Development 2nd edition.
